# Author Correction: Both fallopian tube and ovarian surface epithelium are cells-of-origin for high-grade serous ovarian carcinoma

**DOI:** 10.1038/s41467-026-73799-2

**Published:** 2026-07-23

**Authors:** Shuang Zhang, Igor Dolgalev, Tao Zhang, Hao Ran, Douglas A. Levine, Benjamin G. Neel

**Affiliations:** Laura and Isaac Perlmutter Cancer Center, NYU Langone Health, New York, NY USA

Correction to: *Nature Communications* 10.1038/s41467-019-13116-2,published online 26 November 2019

In the version of this article initially published, due to figure preparation mistakes, the lower-left micrograph in Fig. 8a (olaparib, 0 μM) was an inadvertent duplicate of Fig. 8d (carboplatin, 8 μM), and the error bars for the time points for niraparib and paclitaxel represented s.d. instead of ±SEM. The source data for Fig. 3c were incorrect and did not list the actual width and length measurements and calculated volumes for tumors shown in Fig. 3c. In the source data for Fig. 8, plate reader values used to monitor drug effects were transposed and duplicated from the original files. In Supplementary Fig. 7c, *y*-axis units (0–5 cm^3^) did not list the correct scale (0.0–0.5 cm^3^). Due to the age of the article, the figures and source data cannot be updated directly. Revised figures and source data are available alongside this amendment. This notice serves to correct the article.

## Supplementary information

Corrected Fig. 8

Corrected Supplementary Fig. 7

## Source data

Corrected Source Data

