# Supplementary material for: Both fallopian tube and ovarian surface epithelium are cells-of-origin for high-grade serous ovarian carcinoma
Source: Nat Commun. 2019 Nov 26;10:5367. doi: 10.1038/s41467-019-13116-2 (PMC6879755; doi:10.1038/s41467-019-13116-2)
Supplement: Supplementary file 1 — Supplementary Information [file 41467_2019_13116_MOESM1_ESM.pdf]

# Both Fallopian Tube and Ovarian Surface Epithelium Are Cells-of Origin for High-Grade Serous Ovarian Carcinoma

**Zhang et al.**

# Supplementary Figures and Figure Legends

## Supplementary Fig.1

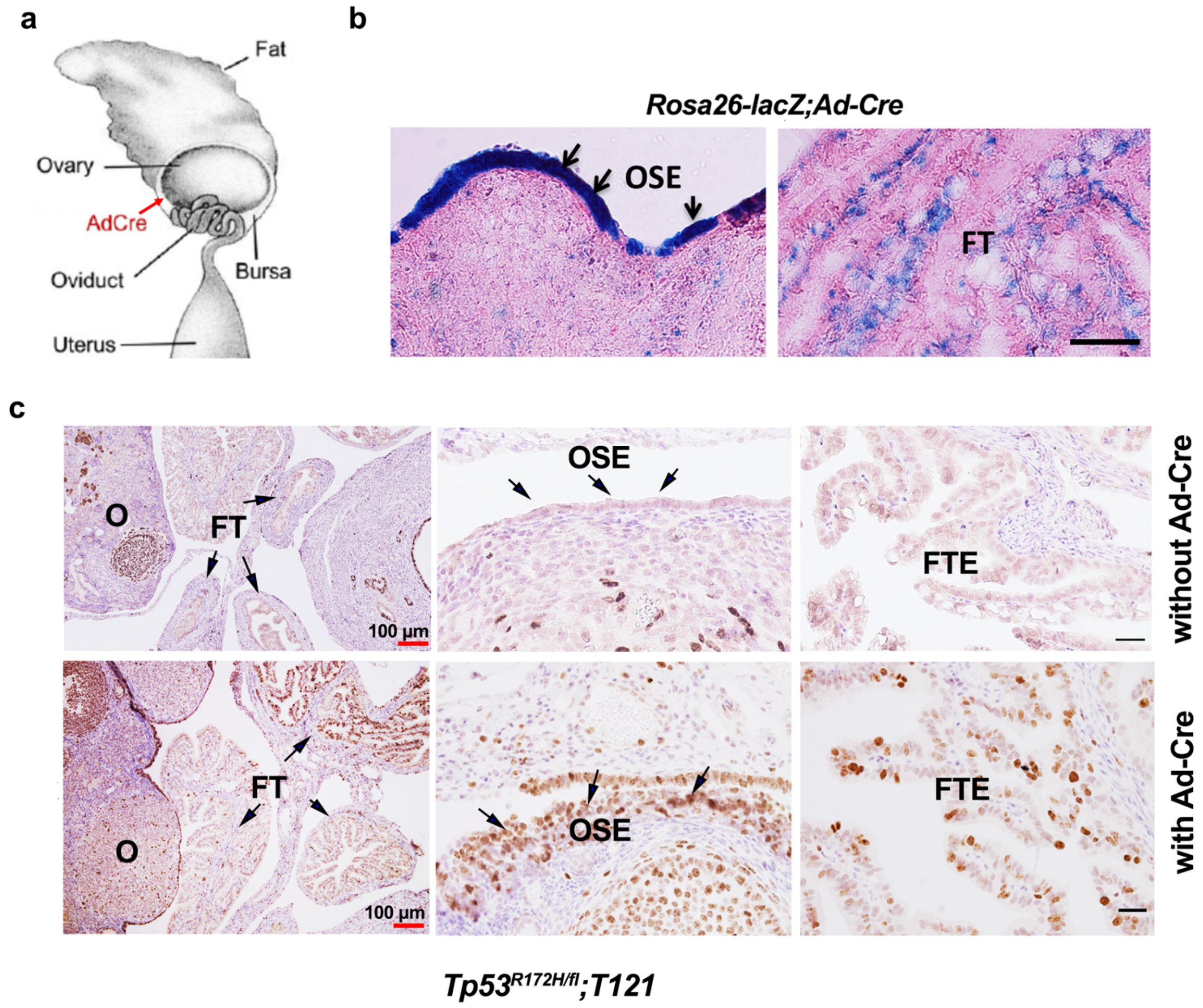


**Supplementary Figure 1. Ad-Cre injection into ovarian bursa targets OSE *and* FTE. a,** Schematic showing bursal injection approach. **b**, X-gal stain shows β-gal expression in OSE (left) and FTE (right) of Ad-Cre-injected *Rosa26-LacZ* female mice. **c,** Representative Ki67 staining (IHC) in *Tp53$^{R172H/fl}$;T121* mice with or without Ad-Cre injection; ovary and oviducts (FT) were collected 1 month after injection; scale bars: 10μm. OSE: ovarian surface epithelium. FTE: fallopian tube epithelium. O: ovary. FT: fallopian tube.

## Supplemetary Fig.2

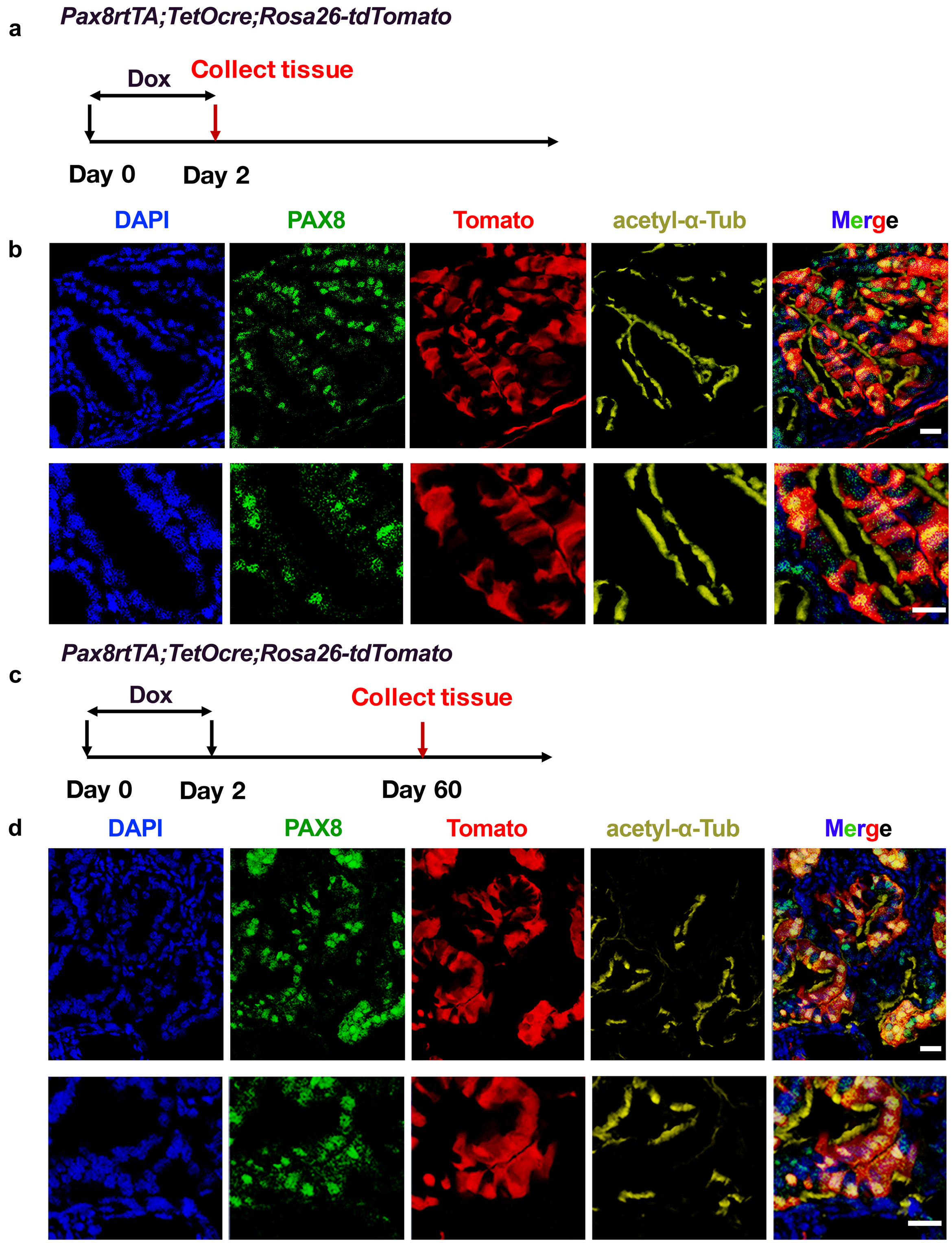

**Supplementary Figure 2. Lineage tracing of *Pax8* cells in mouse fallopian tube a,** Scheme depicting induction of adult *Pax8-rtTA;tetO-Cre;Rosa26-tdTomato* mice by treatment with Dox for 2 days (pulse), followed by sacrifice and tissue collection **b,** Immunofluorescence staining for acetylated-α-tubulin (yellow), PAX8 (green) and DAPI (blue) in mouse oviductal epithelium after 2-day Dox treatment **c,** Scheme showing "chase" experiment **d**, Immunofluorescence staining for acetylated-α-tubulin (yellow) and PAX8 (green) in oviductal epithelium of *Pax8rtTA;TetOcre;Rosa26-tdTomato* mice, 60 days after Dox induction; lower panels in (**b**) and (**d**) are higher magnifications of the corresponding upper panels. DAPI-stained nuclei are shown in blue. Tomato (red), acetylated-α-tubulin (yellow), PAX8 (green) and DAPI (blue) staining, with the overlap (Merge) are shown. Scale bars: 20μm.

## Supplementary Fig.3

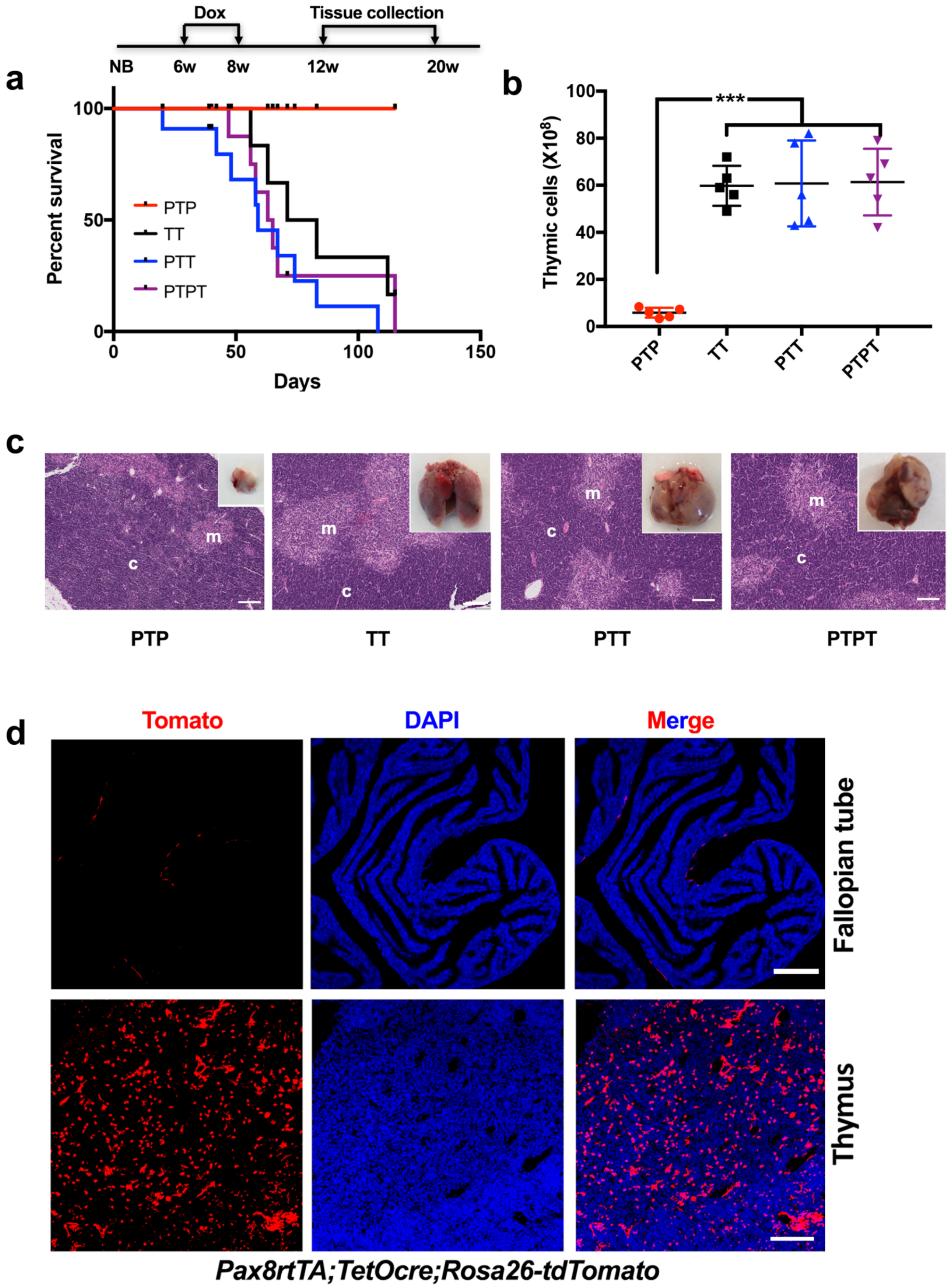

**Supplementary Figure 3. "Leaky" expression of *TetOCre* in thymic epithelium of PTT or PTPT mice results in lethal hyperplasia. a,** Survival curves of PTP, TT, PTT and PTPT mice; TT: *TetOCre;T12;* Source data are provided as a Source Data file. **b,** Thymic cellularity in control, TT, PTT and PTPT mice at 9 weeks (n=5 for each group); data represent means ± SEM, ***P<0.01, Tukey's multiple comparison test. **c,** Representative morphology and H&E stains of thymi from PTP, TT, PTT and PTPT mice; m, medulla; c, cortex **d,** Representative Tomato fluorescence in thymi from *Pax8rtTA;TetOcre;Rosa26-tdTomato* without Dox treatment; slides were counterstained with DAPI. Scale bars: 10 μm.

## Supplementary Fig.4

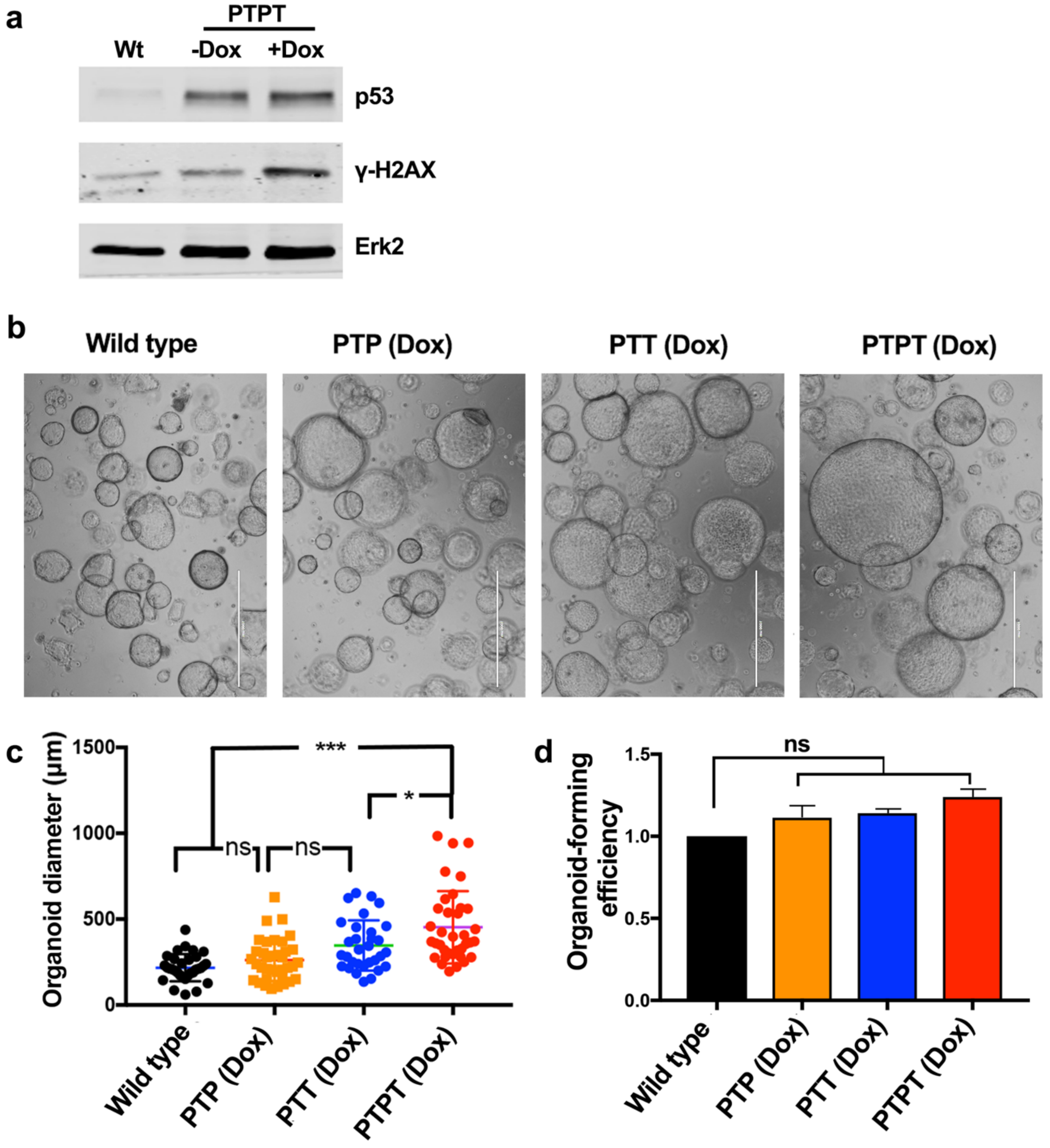


**Supplementary Figure 4. Effect of genotype on FTE organoid size a,** Representative immunoblot of p53 and γ-H2AX in PTPT organoids without Dox treatment and 3 passages after Dox induction; ERK2 serves as a loading control. WT, wild type organoid. **b**, Bright field views of FTE organoids from the indicated mice, cultured for 6 days; each culture was established by seeding 5,000 FTE cells. **c**, Average diameter of organoids with indicated genotypes, measured on day 6 of culture; organoids from 3 wells were analyzed for each group; Source data are provided as a Source Data file. **d**, Organoid-forming efficiency of FTE cells from the indicated genotypes, quantified at day 6 of culture; organoids were derived from 3 independent mice of each genotype (also see Source Data). Data represent mean ± SEM, *P<0.05; ***P<0.01, ns, not significant, Tukey's multiple comparison test.

## Supplementary Fig.5

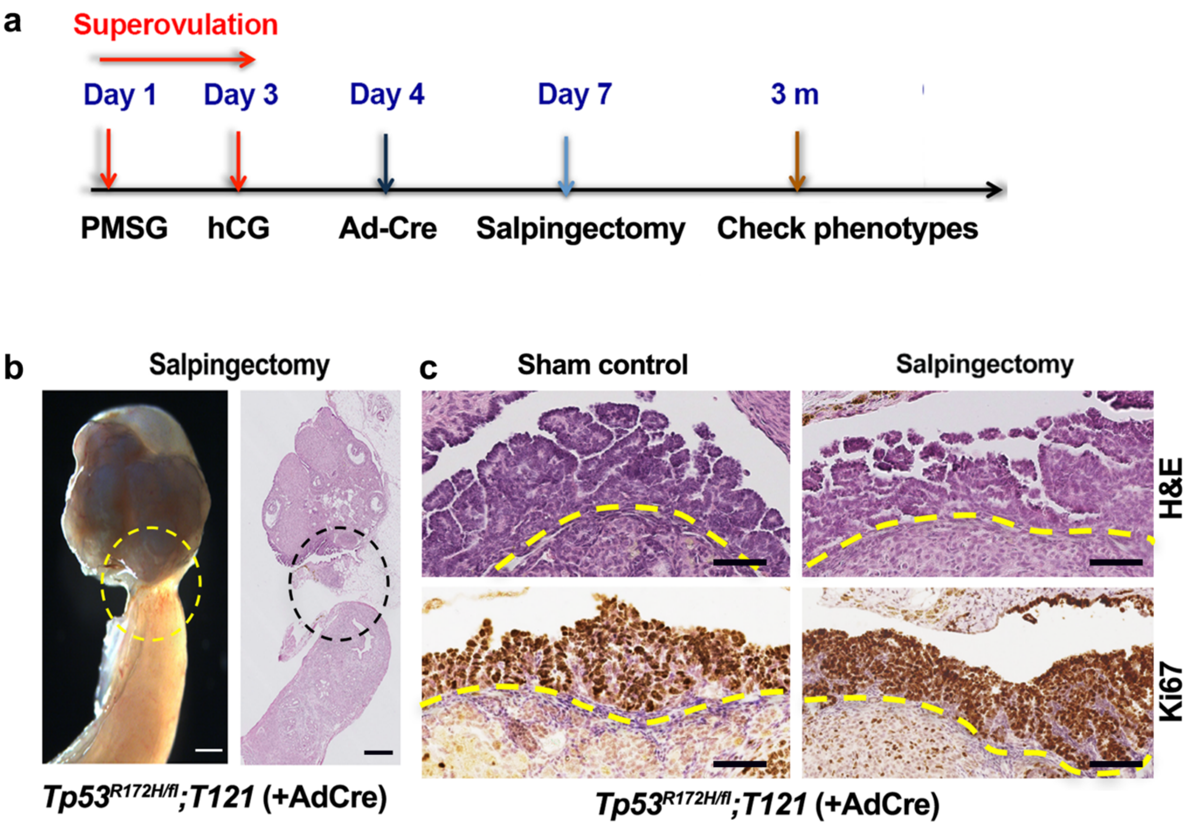


**Supplementary Figure 5. Salpingectomy does not prevent neoplasia in Ad-Cre-injected *Tp53$^{R172H/fl;}$T121* mice. a**, Schematic showing experimental strategy **b**, Micrograph showing gross morphology (Left panel) and H&E-stained section of female genital tract from salpingectomized *Tp53$^{R172H/fl}$;T121* mice, injected with Ad-Cre (Right panel); dashed ovals show absence of fallopian tube. Scale bar:100μm **c,** Representative H&E staining and Ki67 IHC of section from Ad-Cre-injected *Tp53$^{R172H/fl}$;T121* mice with or without (Sham control) salpingectomy, assessed 3 months post-injection; line shows border between tumor and underlying cells. Scale bar:100μm

## Supplementary Fig.6

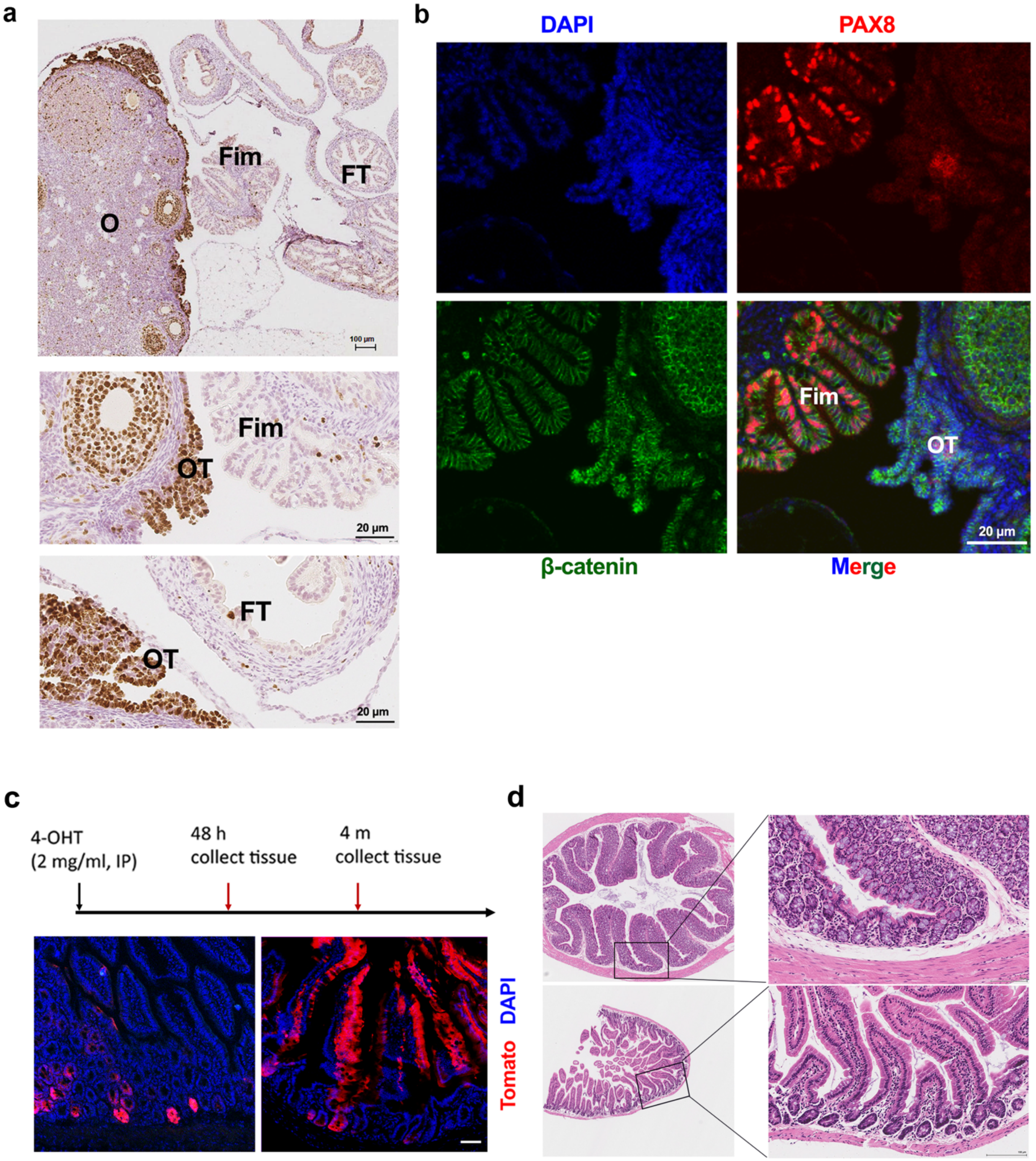


**Supplementary Figure 6.** ***Lgr5-Cre*** **only directs tumorigenesis in OSE. a,** Ki67 IHC staining of OSE and FTE from an LPT mouse; bottom panel is higher magnification (20X) of top image. **b**, PAX8 (red) and β-catenin (green) immunofluorescence in ovary and fallopian tube; DAPI (blue) was used as nuclear counterstain. FTE stains strongly for PAX8, while the ovarian tumor is PAX8-negative; OT: OSE-derived tumor, FT: fallopian tube, Fim: fimbria. **c,** Tomato+ intestinal epithelial clones 48 h or 4 months post 4-OHT induction of *Lgr5-Cre;Rosa26-tdTomato* mice; scale bars, 50µm **d,** H&E-stained intestinal section from LPT mouse, 11 months after 4-OHT treatment; note the absence of carcinoma.

## Supplementary Fig.7

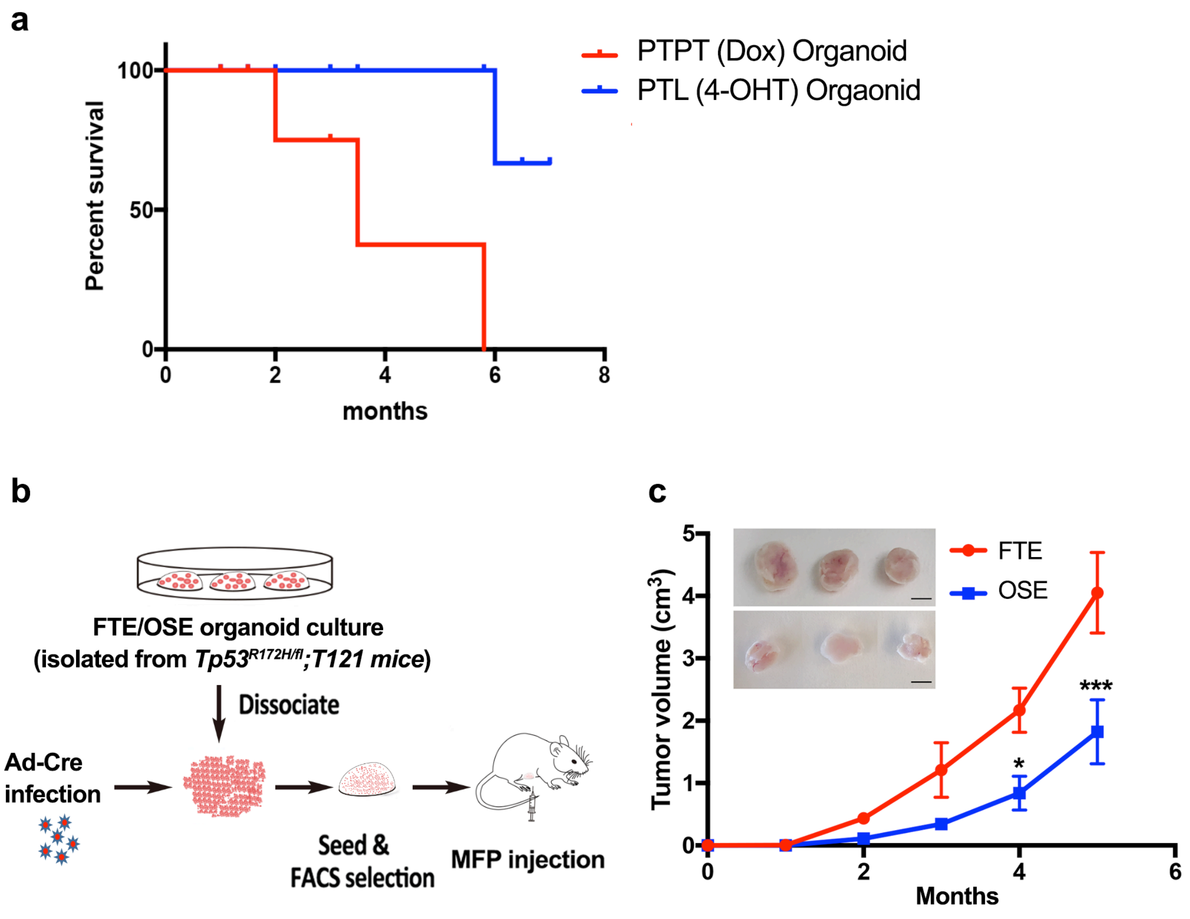


**Supplementary Figure 7. OSE-derived tumors develop with longer latency and at lower penetrance than those from FTE. a,** Survival curves of mice injected orthotopically with PTPT-derived FTE organoids and PTL-derived OSE organoids ($10^5$ cells each) **b,** Schematic shows experimental strategy for mammary fat pad (MFP) injection experiments. **c,** Average tumor volumes in MFPs of mice, monitored over 6 months post-injection of $10^5$ cells from OSE or FTE organoids derived from *Tp53*$^{R172H/fl}$;*T121* mice, as indicated; data represent mean ± SEM, P<0.5, ***P<0.001, 2-way ANOVA; Source data are provided as a Source Data file.

## Supplementary Fig.8

a

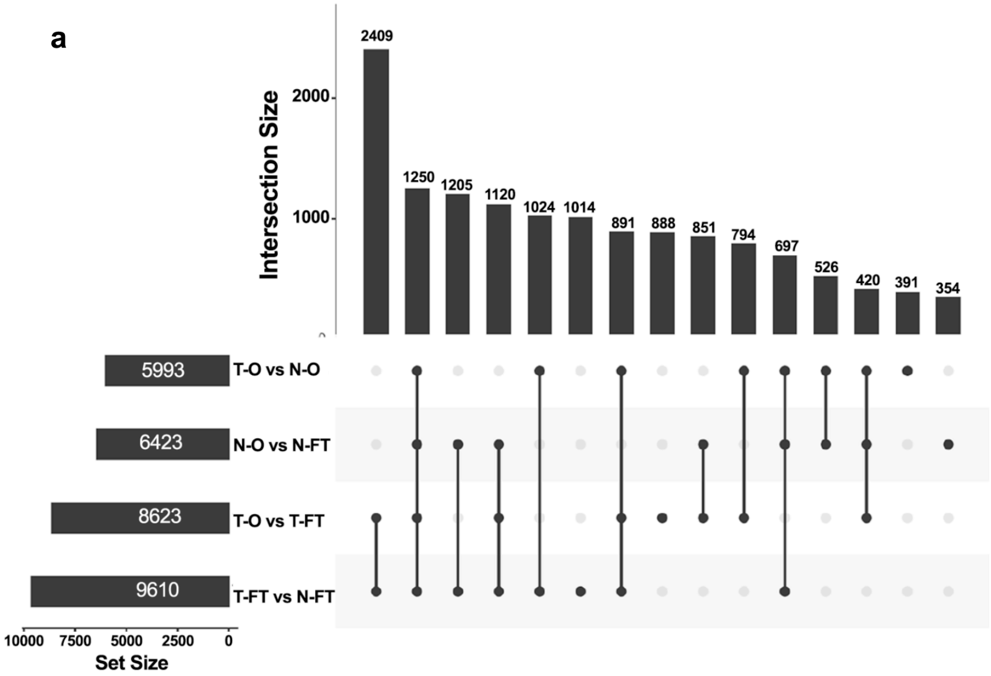


b

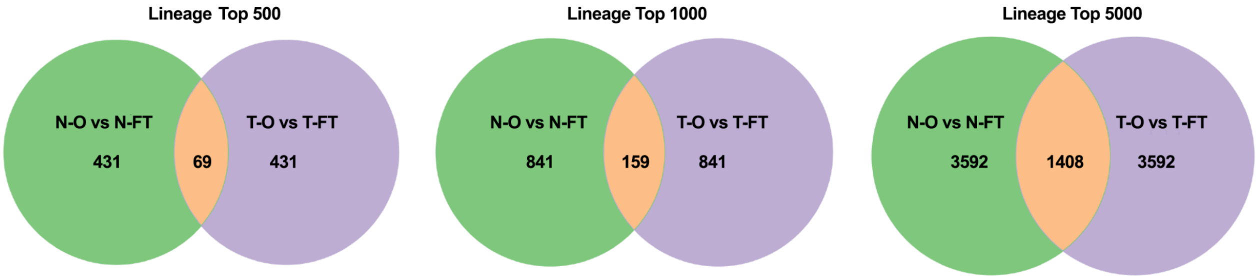


**Supplementary Figure 8. Contribution of lineage-dependent and -independent gene expression to differential gene expression in FTE- vs OSE-derived tumors a,** UpSet diagram showing overlaps of differentially expressed genes ($P_{adj}$ <0.05) in T-FT vs N-FT, N-O vs N-FT, T-O vs N-O, and T-O vs T-FT groups. Source data are provided as a Source Data file. **b,** Venn diagrams showing overlaps of the top 500, 1,000, and 5,000 most differentially expressed genes between the indicated groups.

## Supplementary Fig.9

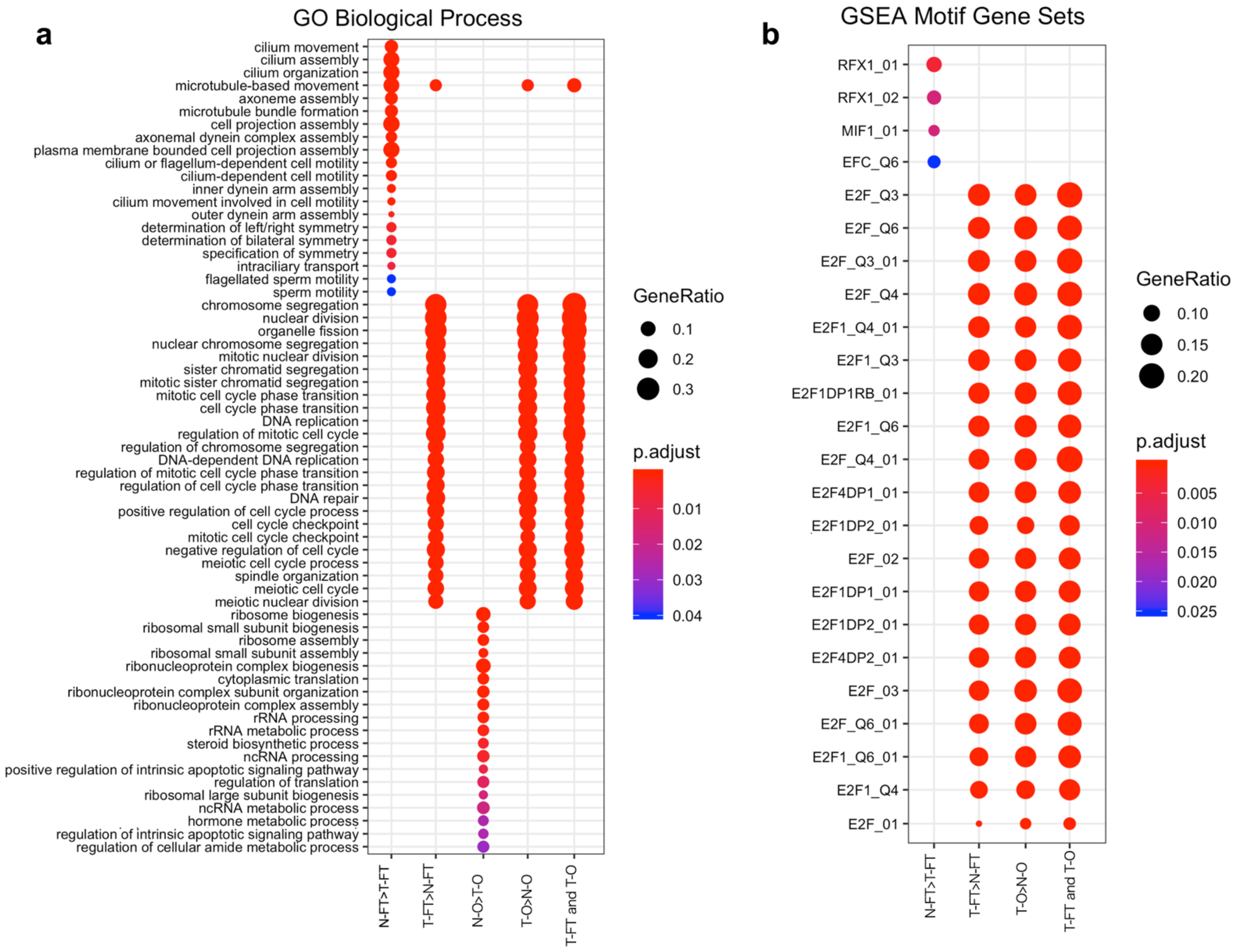


**Supplementary Figure 9. Pathway analyses comparing normal FTE and OSE and cognate tumors a,** GO biological processes **b,** GSEA motif gene sets; size of each circle represents the number of DEGs within each category; color indicates significance level.

## Supplementary Fig.10

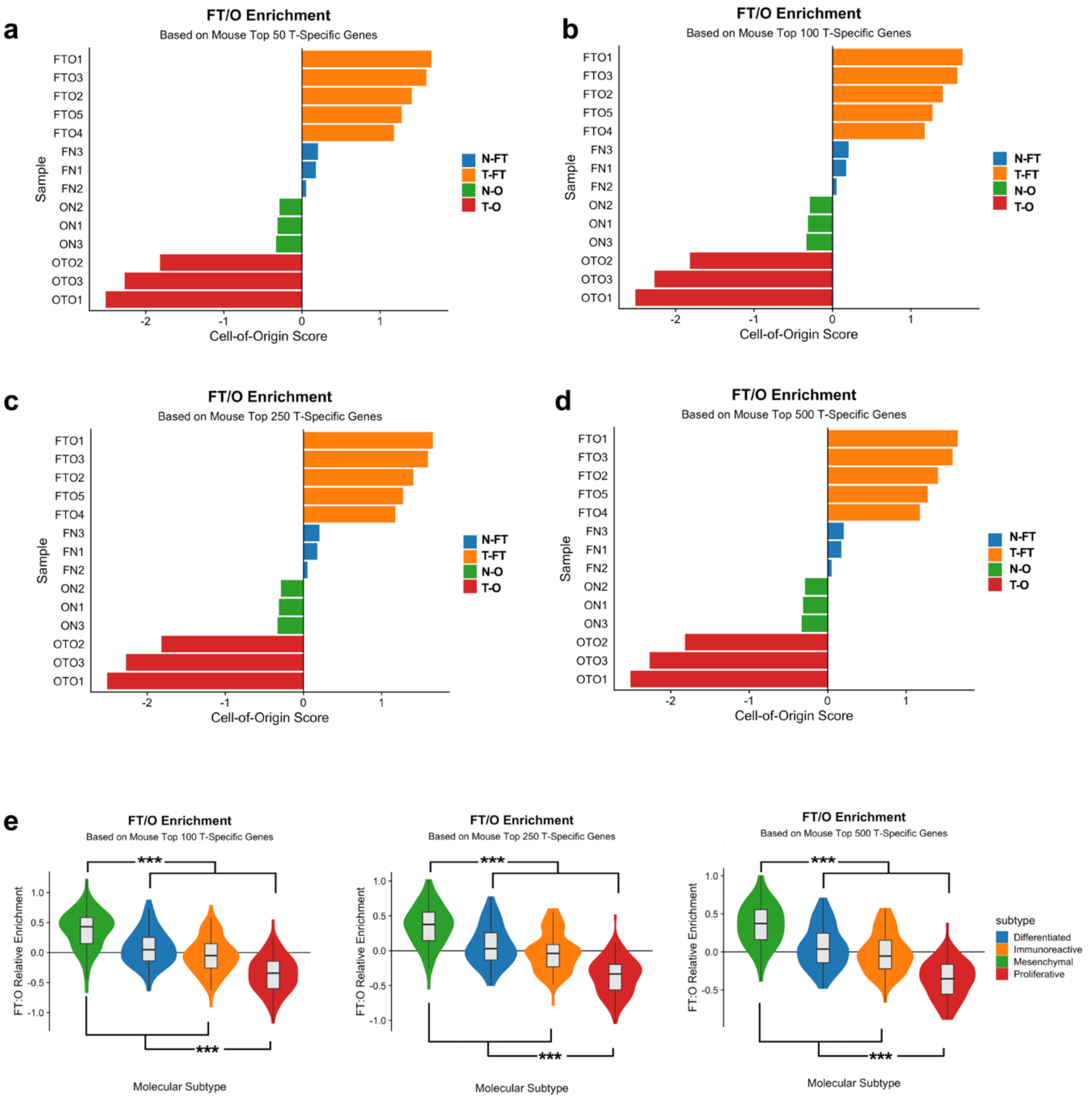


**Supplementary Figure 10. Application of cell-of-origin scores to TCGA data** Signatures were developed based on the top 50 (**a**), 100 (**b**), 250 (**c**), or 500 (**d**) FTE- or OSE-derived tumor-specific genes, and compared with the mean scaled gene expression from normal FTE (N-FT), normal OSE (N-O), and FTE (T-FT)- or OSE (T-)-derived tumors, as indicated. See Methods for details. **e,** Application of cell-of-origin score, calculated based on the mouse top 100, 250, or 500 DEGs, as indicated, to TCGA samples. Colors indicate the transcriptional subtype assigned to each sample by TCGA, ***P < 0.001, Wilcoxon rank sum test. See Figure 7e and Methods for details, also see source data for P values.

## Supplementary Fig.11

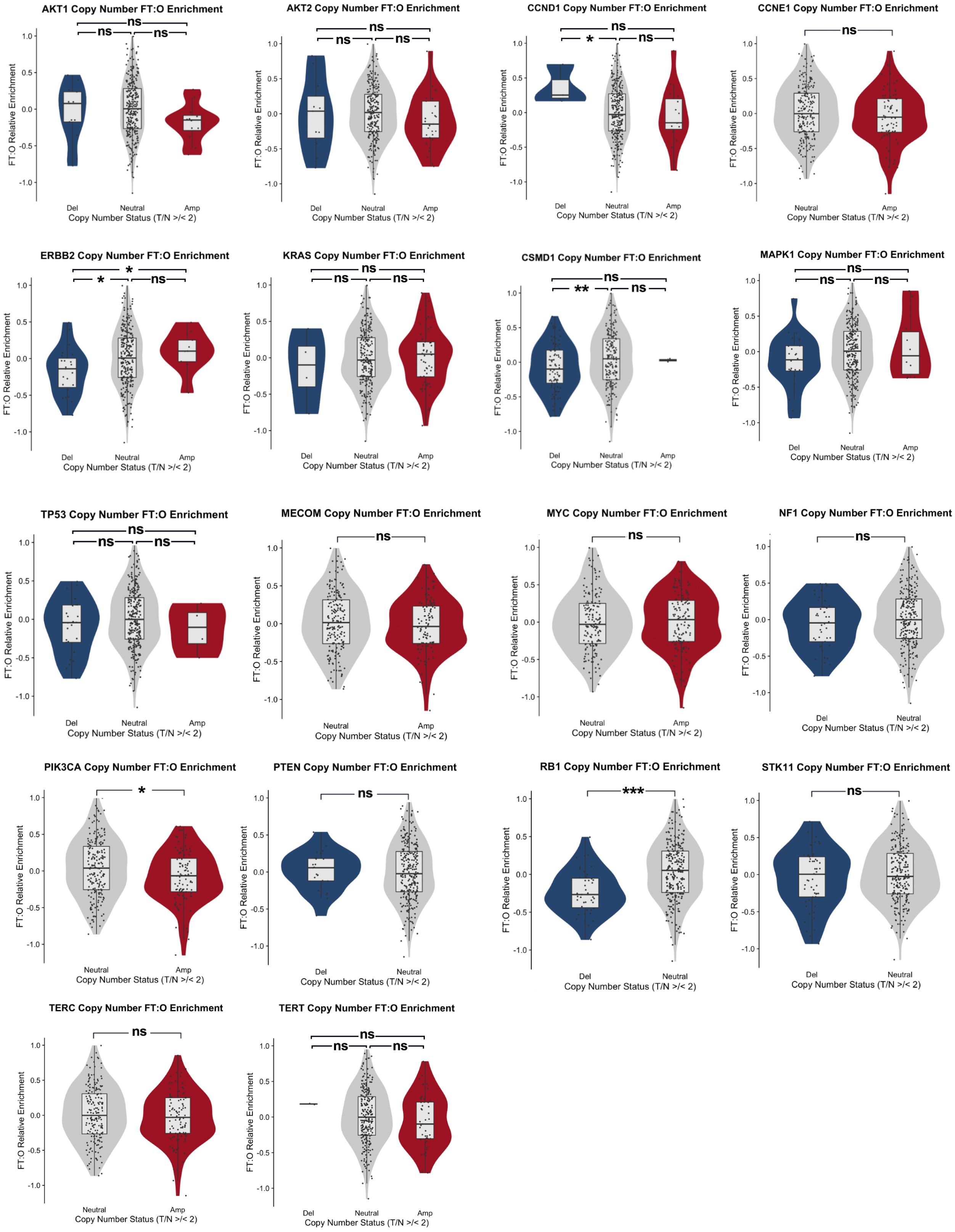

**Supplementary Figure 11. Relationship between copy number landscape and cell-of-origin score in TCGA samples** Cell-of-origin (FT:O) score (based on top 50 DEGs) was calculated for all evaluable TCGA samples and compared with the copy number status of frequently altered genes. Note that essentially all major copy number abnormalities (CNAs) are found in both OSE- and FTE-like tumors, although there some CNAs might be more frequent in OSE-derived, compared with FTE-like, tumors (Source data are provided as a Source Data file). Amp: T:N>2; Del: T:N<0.5, ns, not significant, *P<0.05, **P<0.01, ***P<.001, Wilcoxon rank sum test..

## Supplementary Fig.12

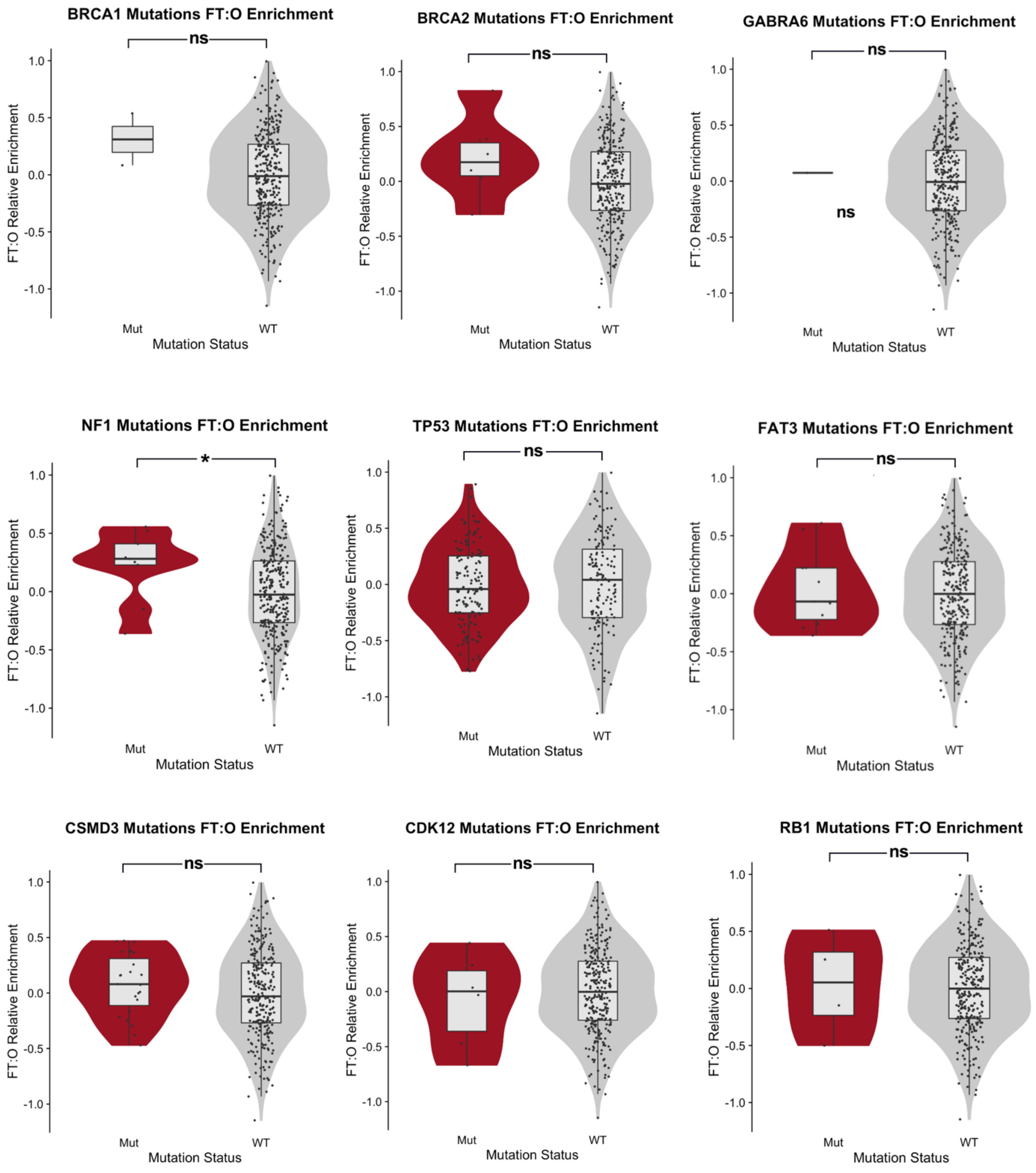


**Supplementary Figure 12. Relationship between mutational landscape and cell-of-origin score in TCGA samples** Cell-of-origin (FT:O) score (calculated based on top 50 DEGs) was calculated for all evaluable TCGA samples and compared with the mutational status of frequently altered genes. Essentially all genes with significant numbers of somatic mutations in TCGA can be found in an OSE- or FTE-like tumors, although there might be enrichment for specific mutations in FTE-derived, compared with OSE-like tumors (also see source data for P values), ns, not significant, *P<0.05.

## Supplementary Fig.13

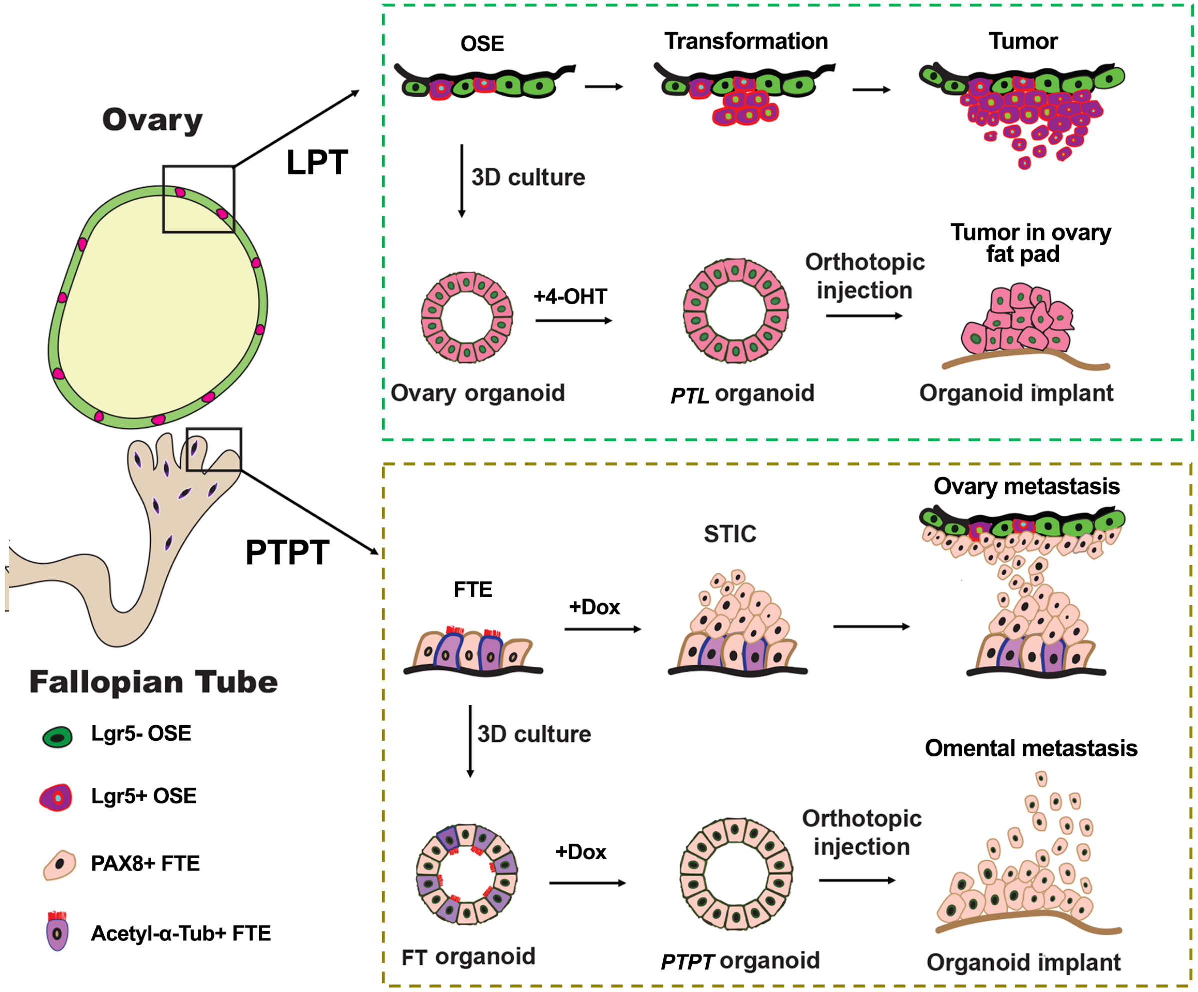


**Supplementary Figure 13. Model showing approaches used to assess HGSOC cell-of-origin** Combined RB family inactivation (via T121 expression) and *Tp53* mutation in *Lgr5+* OSE cells, initiated by CRE activation (with 4-OHT), results in patchy areas of OSE transformation that expand, taking over the entire ovarian surface and eventually spreading to the peritoneal cavity (**upper panel)**. These processes can be reproduced by transplanting cognate OSE organoids. **Lower panel**: The same genetic events also cause transformation of *Pax8+* FTE, leading to Serous Tubal Intraepithelial Carcinoma (STIC) and ovarian metastasis. These behaviors can be recapitulated in FTE organoids, which generate widespread abdominal metastasis following orthotropic injection.

## Supplementary Methods

### Primer sequences used for genotyping

*Tp53 fl*: Genotyping floxed forward: 5'-CAC AAA AAC AGG TTA AAC CCA G-3'; Genotyping floxed reverse: 5'-AGC ACA TAG GAG GCA GAG AC-3';

*Pax8-rtTA*: Genotyping forward: 5'-CCA TGT CTA GAC TGG ACA AGA-3'; Genotyping reverse: 5'-CTC CAG GCC ACA TAT GAT TAG-3'

*TetO-Cre*: Genotyping forward: 5'-GCG GTC TGG CAG TAA AAA CTA TC-3'; Genotyping reverse: 5'-GTG AAA CAG CAT TGC TGT CAC TT-3'

*T121:* Genotyping floxed forward: 5'-GAA TCT TTG CAG CTA ATG GAC C-3'; Genotyping floxed reverse: 5'-GCA TCC CAG AAG CTC CAA AG-3'

*Tp53 R172H:* Genotyping floxed forward: 5'-ACC TGT AGC TCC AGC ACT GG-3'; Genotyping floxed reverse: 5'-ACA AGC CGA GTA ACG ATC AGG-3'

*Lgr5-Cre:* Genotyping forward: 5'-GGC GCG GCA ACA CCA TTT TT-3'; Genotyping reverse: 5'-TCC GGG CTG CCA CGA CCA A-3'
