# Supplementary material for: Both fallopian tube and ovarian surface epithelium are cells-of-origin for high-grade serous ovarian carcinoma
Source: Nat Commun. 2019 Nov 26;10:5367. doi: 10.1038/s41467-019-13116-2 (PMC6879755; doi:10.1038/s41467-019-13116-2)
Supplement: Supplementary file 3 — Reporting Summary [file 41467_2019_13116_MOESM3_ESM.pdf]

nature research

Corresponding author(s): Benjamin G. Neel

Last updated by author(s): Sep 27, 2019

Reporting Summary

Nature Research wishes to improve the reproducibility of the work that we publish. This form provides structure for consistency and transparency in reporting. For further information on Nature Research policies, see Authors & Referees and the Editorial Policy Checklist .

Statistics

For all statistical analyses, confirm that the following items are present in the figure legend, table legend, main text, or Methods section.

n/a	Confirmed	
☐	☒	The exact sample size (*n*) for each experimental group/condition, given as a discrete number and unit of measurement
☐	☒	A statement on whether measurements were taken from distinct samples or whether the same sample was measured repeatedly
☐	☒	The statistical test(s) used AND whether they are one- or two-sided<br>*Only common tests should be described solely by name; describe more complex techniques in the Methods section.*
☒	☐	A description of all covariates tested
☐	☒	A description of any assumptions or corrections, such as tests of normality and adjustment for multiple comparisons
☐	☒	A full description of the statistical parameters including central tendency (e.g. means) or other basic estimates (e.g. regression coefficient) AND variation (e.g. standard deviation) or associated estimates of uncertainty (e.g. confidence intervals)
☐	☒	For null hypothesis testing, the test statistic (e.g. *F*, *t*, *r*) with confidence intervals, effect sizes, degrees of freedom and *P* value noted<br>*Give P values as exact values whenever suitable.*
☒	☐	For Bayesian analysis, information on the choice of priors and Markov chain Monte Carlo settings
☒	☐	For hierarchical and complex designs, identification of the appropriate level for tests and full reporting of outcomes
☐	☒	Estimates of effect sizes (e.g. Cohen's *d*, Pearson's *r*), indicating how they were calculated

Our web collection on statistics for biologists contains articles on many of the points above.

Software and code

Policy information about availability of computer code

Data collection	No software was used for data collection beyond that supplied with machines specified in methods.
Data analysis	GrapPad Prism 8 (https://www.graphpad.com/scientific-software/prism/) was used for surviving analyses, tumor growth analyses, organoid forming efficiency, cell growth curves, dose-response curves. Flow Jo was used to gate the GFPhi and GFPneg cells. Fiji (Imagine J) was used to measure the tissue colonies, hyperplasia areas, organoid numbers, sizes and invasions. Illumina bcl2fastq software was used to demultiplex and converted to FASTQ format using. The reads were adapter and quality trimmed with Trimmomatic and then aligned to the mouse genome (build mm10/GRCm38) using the splice-aware STAR aligner. The featureCounts program was utilized to generate counts for each gene based on how many aligned reads overlap its exons. The counts were normalized and used to test for differential expression using negative binomial generalized linear models implemented by the DESeq2 R package. Statistical analysis and visualization of gene sets were performed using the clusterProfiler R package. TCGA Ovarian Cancer batch effects normalized mRNA data, somatic mutations, tumor gene-level copy number data, PARADIGM pathway activity, and molecular subtypes were retrieved from the UCSC Xena Pan-Cancer Atlas hub.

For manuscripts utilizing custom algorithms or software that are central to the research but not yet described in published literature, software must be made available to editors/reviewers. We strongly encourage code deposition in a community repository (e.g. GitHub). See the Nature Research guidelines for submitting code & software for further information.

Data

Policy information about availability of data

All manuscripts must include a data availability statement. This statement should provide the following information, where applicable:

- Accession codes, unique identifiers, or web links for publicly available datasets
- A list of figures that have associated raw data
- A description of any restrictions on data availability

NA sequence data have been deposited in the GEO database under accession code GSE125016. TTCGA ovarian cancer batch effects-normalized mRNA data, somatic mutations, tumor gene-level copy number data, PARADIGM pathway activity, and molecular subtypes referenced during the study are available in a public repository from the UCSC Xena Pan-Cancer Atlas hub. The data underlying Figs 2f, h, i, 3c, 5e, f, 6c, d, f, g, 7b, e, 8 and Supplementary Figs 3a, b, 4a, b, c, 7a, c, 8a, 10e, 11, 12 are provided as a Source Data file. All other data supporting the findings of this study are available within the article, its supplementary information files, or from the corresponding author upon reasonable request. A reporting summary for this article is available as a Supplementary Information file.

Field-specific reporting

Please select the one below that is the best fit for your research. If you are not sure, read the appropriate sections before making your selection.

[x] Life sciences [ ] Behavioural & social sciences [ ] Ecological, evolutionary & environmental sciences

For a reference copy of the document with all sections, see nature.com/documents/nr-reporting-summary-flat.pdf

Life sciences study design

All studies must disclose on these points even when the disclosure is negative.

Sample size	Sample sizes and statistical tests for each experiment are denoted in the figure legends.
Data exclusions	No data were excluded from the analyses
Replication	All results in the manuscript were replicated by using independent mice with the same genotypes, as indicated in the text, figure legends and/or methods.
Randomization	This is not relevant for the study, all data were detected on transgenic mice , xenografts (orthotopic or mammary fat pad injections) or organoids with specified genotypes.
Blinding	The investigator was not blinded to group allocation in this study. Blinding was not necessary because the experiment involved mice of different genotypes, which were investigator determined.

Reporting for specific materials, systems and methods

We require information from authors about some types of materials, experimental systems and methods used in many studies. Here, indicate whether each material, system or method listed is relevant to your study. If you are not sure if a list item applies to your research, read the appropriate section before selecting a response.

Materials & experimental systems

n/a	Involved in the study	
[ ]	[x]	Antibodies
[ ]	[x]	Eukaryotic cell lines
[x]	[ ]	Palaeontology
[ ]	[x]	Animals and other organisms
[x]	[ ]	Human research participants
[x]	[ ]	Clinical data

Methods

n/a	Involved in the study	
[x]	[ ]	ChIP-seq
[ ]	[x]	Flow cytometry
[x]	[ ]	MRI-based neuroimaging

Antibodies

Antibodies used	Primary antibodies for (IHC) included: Ki67 1:200 (ab15580, Abcam), γ-H2AX 1:500 (05-636, Thermo Fisher Scientific), CK7 1:200

(ab181598, Abcam), Stathmin-1 1:200 (3352S, cell signaling), P16 1:200 (sc-1661, Santa Cruz), PAX8 1:200 (10336-1-AP, Proteintech), p53 1:800 (P53-CM5P-L, Leica). Secondary antibodies included: goat anti-chicken IgY-HRP 1:200 (sc-2428, Santa Cruz), goat anti-rabbit IgG-HRP 1:200 (sc-2030, Santa Cruz).
Primary antibodies (IF) were: GFP 1:300 (ab13970, Abcam), WT1 1:200 (ab15249, Abcam), E-cadherin 1:200 (ab15148, Abcam). Secondary antibodies included: goat anti-mouse IgG, Alexa Fluor® 647 conjugate 1:200 (A28181, Thermo Fisher Scientific), goat anti-rabbit IgG, Alexa Fluor® 555 conjugate 1:200 (A27039, Thermo Fisher Scientific), and goat anti-chicken IgY H&L (Alexa Fluor® 488) 1:200 (ab150169, Abcam).

Validation

All the primary antibodies including Ki67 (https://www.abcam.com/ki67-antibody-epr3610-alexa-fluor-488 ab197234.html?gclid=CjwKCAiA99vhBRBnEiwAwpk-uDmNh_PmNQSc7bSDrlPhCRsjMrSCzqUqhxqFFAAaSa1hZGXQtg3p3xoCwYkQAvD_BwE),
γ-H2AX (http://www.emdmillipore.com/US/en/product/Anti-phospho-Histone-H2A.X-Ser139-Antibody-clone-JBW301,MM_NF-05-636),
CK7 (https://www.abcam.com/cytokeratin-7-antibody-epr17078-ab181598.html),
Stathmin-1 (https://www.cellsignal.com/products/primary-antibodies/stathmin-antibody/3352),
P16 (http://www.antibodyreview.com/products/559100.0/p16-Antibody-(F-12)-Santa-Cruz-Biotechnology-sc-1661.html),
PAX8 (https://www.ptglab.com/products/PAX8-Antibody-10336-1-AP.htm),
p53(https://www.leicabiosystems.com/fileadmin/img_uploads/novocastra_reagents/Novocastra_datasheets/p53-cm5p-l.pdf) used in the manuscript have specie reactivity on mouse and applicable for IHC/IF.
The Anti-SV40 T Antigen (Ab-1) Mouse mAb (http://www.emdmillipore.com/US/en/product/Anti-SV40-T-Antigen-Ab-1-Mouse-mAb-PAb419,EMD_BIO-DP01) has specie reactivity on mouse and applicable for immunoblot. The validation statements, relevant citations and antibody profiles can be found on the manufacture's website in online databases through the links.

Eukaryotic cell lines

Policy information about cell lines

Cell line source(s)

FTE cells were isolated from the fallopian tube of wild type, TPT or PTPT mice as indicated. OSE cells were isolated from the ovarian surface epithelium of Lgr5–EGFP–ires–CreERT2 mice, LT or LPT mice as indicated. All cell lines were generated as part of this manuscript. No other cell liens were used.

Authentication

N/A

Mycoplasma contamination

All cell lines were mycoplasma negative as assessed by IDEXX bioresearch (Case # 10168-2018)

Commonly misidentified lines
(See ICLAC register)

N/A

Animals and other organisms

Policy information about studies involving animals; ARRIVE guidelines recommended for reporting animal research

Laboratory animals

Rosa26-tdTomato [B6;129S6-Gt(ROSA)26SORtm9(CAG-tdTomato)Hze], [B6;129-Gt(ROSA)26SorTM1sor], Lgr5-Cre [B6.129P2-Lgr5tm1(cre/ERT2)Cle], Tp53R172H [B6.129S4(Cg)-Trp53 tm2.1Tyj], Trp53flox/flox [FVB;129-Trp53tm1Brn] and nu/nu [NU/J] mice were obtained from the Jackson Laboratory. Conditional TgK18GT121tg/+ BAC transgenic mice (T121 mice) were described previously. Pax8rtTA and TetOcre strains were described previously. Tp53R172H, Trp53flox/flox, T121 mice were interbred with Pax8rtTA and TetOcre mice to obtain Pax8rtTA;TetOcre;Tp53R172H/fl (PTP), Pax8rtTA;TetOcre;T121 (PTT) and Pax8rtTA; TetOcre; Tp53 R172H/fl;T121 (PTPT) mice, respectively. Trp53R172H, Trp53flox/flox, T121 and Lgr5CreERT2 were interbred to obtain Lgr5Cre;Tp53 R172H/fl (LP), Lgr5Cre;T121(LT), Lgr5Cre; Trp53R172H/fl;T121 (LPT) mice, respectively. Rosa26-lacz and Rosa26-tdTomato mice were bred to Pax8rtTA and TetOcre strains to obtain Pax8rtTA;TetOcre;Rosa26-LacZ and Pax8rtTA;TetOcre;Rosa26-tdTomato mice, respectively. When indicated, female mice were euthanized by CO2 inhalation and FT and/or ovaries were harvested for histology and organoid culture.

Wild animals

N/A

Field-collected samples

N/A

Ethics oversight

All animal experiments were approved by, and conducted in accordance with the procedures of, the IACUC at New York University School of Medicine (Protocol no.170602).

Note that full information on the approval of the study protocol must also be provided in the manuscript.

Flow Cytometry

Plots

Confirm that:

- [x] The axis labels state the marker and fluorochrome used (e.g. CD4-FITC).
- [x] The axis scales are clearly visible. Include numbers along axes only for bottom left plot of group (a 'group' is an analysis of identical markers).
- [x] All plots are contour plots with outliers or pseudocolor plots.
- [x] A numerical value for number of cells or percentage (with statistics) is provided.

Methodology

Sample preparation	Ovaries from Lgr5-Cre (Lgr5–EGFP–ires–CreERT2) females (6-8 weeks) were digested as described above and recovered OSE cells were passed through a strainer (40 um) to obtain single-cell suspensions. OSE cells were pelleted by centrifugation at 1,000 g for 5 min and resuspended in PBS containing 2% FBS, Rock inhibitor (Y-27632, 10 uM, STEMCELL Technologies Inc.), DAPI (1ug/ml).
Instrument	MoFloTM XDP
Software	Flow Jo
Cell population abundance	Sorted GFPhi and GFPneg cells were seeded immediately after sorting, the GFP signal is visible from GFPhi population under microscope.
Gating strategy	EGFPhi and EGFPneg cells were gated within the main population of live cells. The gating strategy is supplied in the main figure (Figure 6), not the Supplementary Information.

- [ ] Tick this box to confirm that a figure exemplifying the gating strategy is provided in the Supplementary Information.
