# Supplementary figures and images for: Author Correction: Both fallopian tube and ovarian surface epithelium are cells-of-origin for high-grade serous ovarian carcinoma
Source: Nat Commun. 2026 Jul 23;17:7115. doi: 10.1038/s41467-026-73799-2 (PMC13396802; doi:10.1038/s41467-026-73799-2)

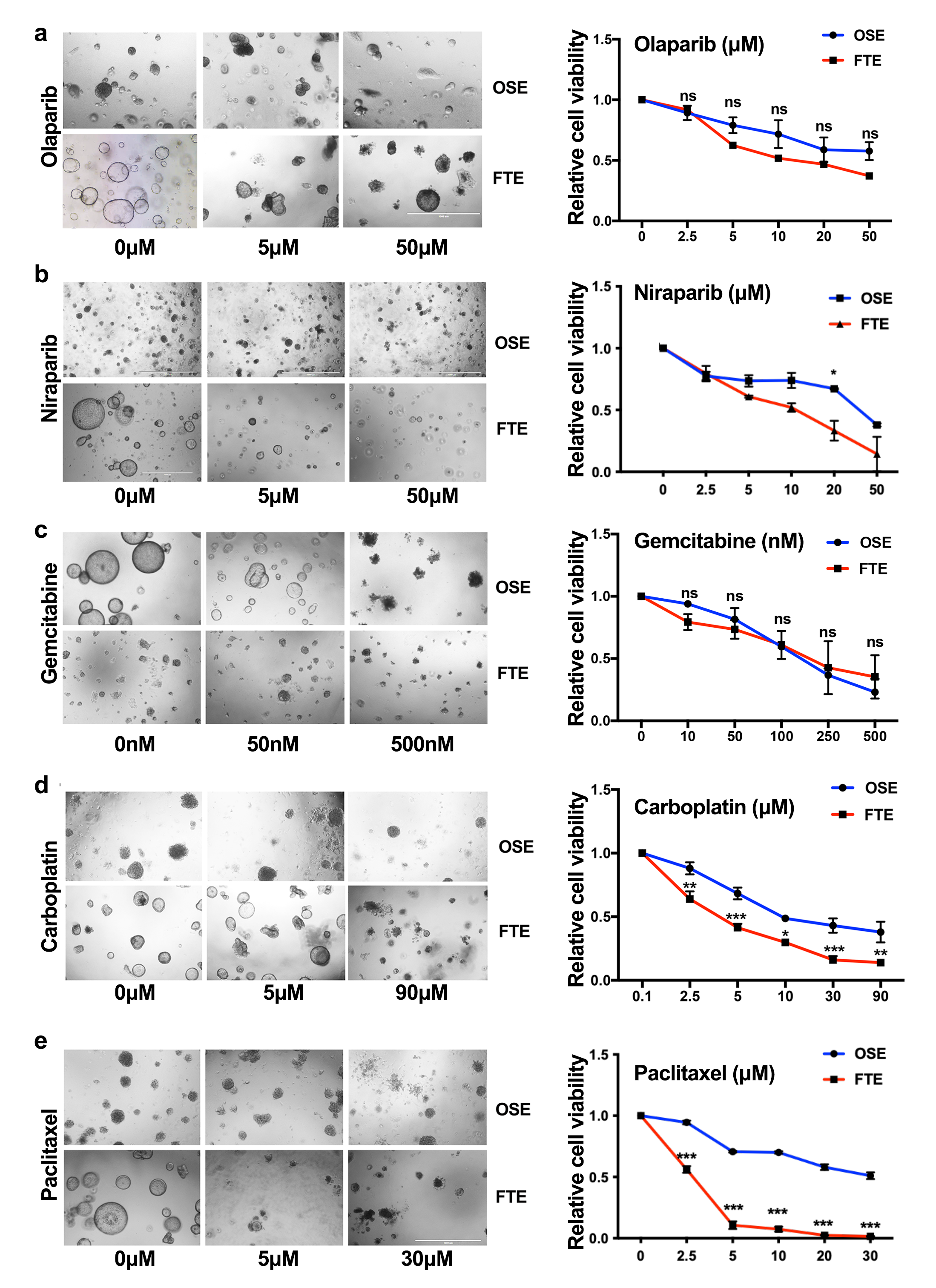

Supplement: Supplementary file 1 — Corrected Fig. 8 [file 41467_2026_73799_MOESM1_ESM.png]
