# Supplementary material for: Author Correction: Both fallopian tube and ovarian surface epithelium are cells-of-origin for high-grade serous ovarian carcinoma
Source: Nat Commun. 2026 Jul 23;17:7115. doi: 10.1038/s41467-026-73799-2 (PMC13396802; doi:10.1038/s41467-026-73799-2)
Supplement: Supplementary file 2 — Corrected Supplementary Fig. 7 [file 41467_2026_73799_MOESM2_ESM.pdf]

Supplementary Fig.7

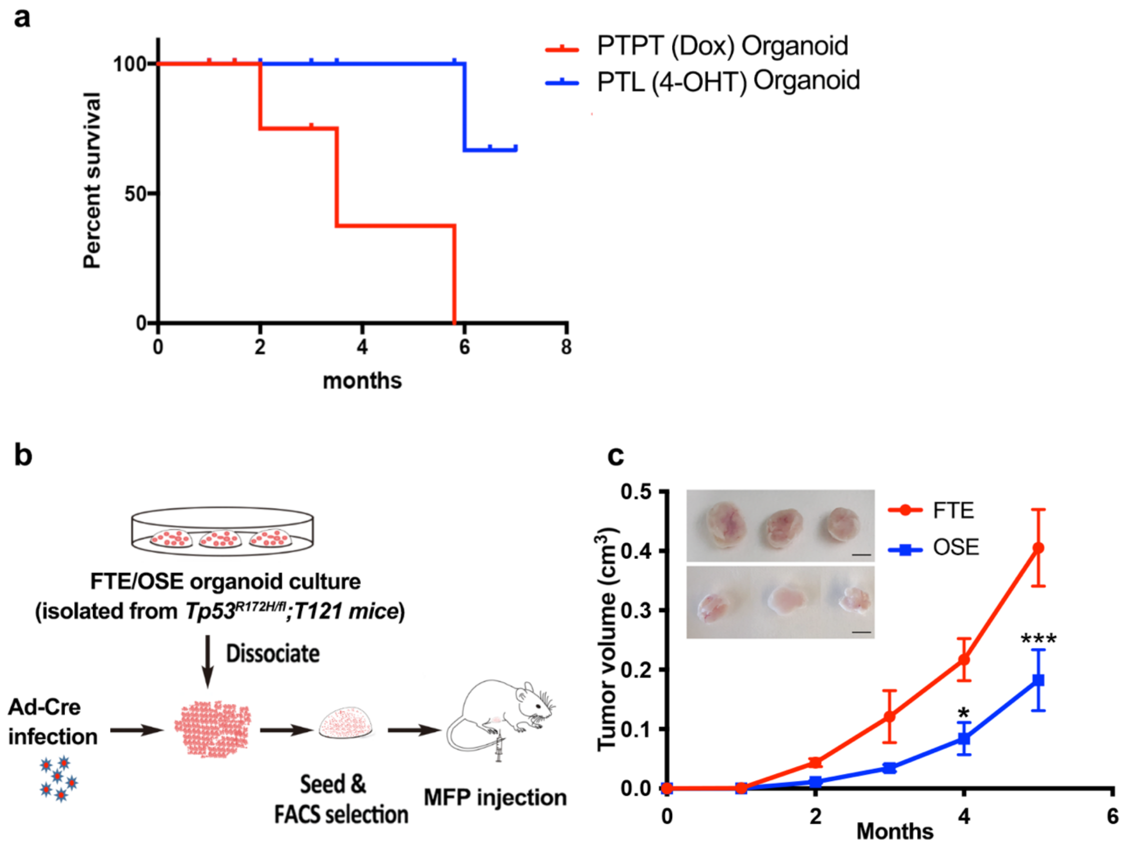

Supplementary Figure 7. OSE-derived tumors develop with longer latency and at lower penetrance than those from FTE. a, Survival curves of mice injected orthotopically with PTPT-derived FTE organoids and PTL-derived OSE organoids (10^5 cells each) **b,** Schematic shows experimental strategy for mammary fat pad (MFP) injection experiments. **c,** Average tumor volumes in MFPs of mice, monitored over 6 months post-injection of 10^5 cells from OSE or FTE organoids derived from *Tp53R172H/fl;T121* mice, as indicated; data represent mean ± SEM, P<0.5, ***P<0.001, 2-way ANOVA; Source data are provided as a Source Data file.
